# Room-Based Assessment of Mobile Air Cleaning Devices Using a Bioaerosol Challenge

**DOI:** 10.1089/apb.2022.0028

**Published:** 2023-03-06

**Authors:** Alan Beswick, Jodi Brookes, Iwona Rosa, Claire Bailey, Charlotte Beynon, Stephen Stagg, Neil Bennett

**Affiliations:** ^1^ Health and Safety Executive Science and Research Centre, Buxton, United Kingdom.; ^2^ The Animal & Plant Health Agency, Addlestone, United Kingdom.

**Keywords:** air cleaner, purification, HEPA, UV-C, bioaerosol, bacteriophage

## Abstract

**Introduction:**

The widespread transmission of the SARS-CoV-2 virus has increased scientific and societal interest in air cleaning technologies, and their potential to mitigate the airborne spread of microorganisms. Here we evaluate room scale use of five mobile air cleaning devices.

**Methods:**

A selection of air cleaners, containing high efficiency filtration, was tested using an airborne bacteriophage challenge. Assessments of bioaerosol removal efficacy were undertaken using a decay measurement approach over 3 h, with air cleaner performance compared with bioaerosol decay rate without an air cleaner in the sealed test room. Evidence of chemical by-product emission was also checked, as were total particle counts.

**Results:**

Bioaerosol reduction, exceeding natural decay, was observed for all air cleaners. Reductions ranged between devices from <2-log per m^3^ room air for the least effective, to a >5-log reduction for the most efficacious systems. One system generated detectable ozone within the sealed test room, but ozone was undetectable when the system was run in a normally ventilated room. Total particulate air removal trends aligned with measured airborne bacteriophage decline.

**Discussion:**

Air cleaner performance differed, and this could relate to individual air cleaner flow specifications as well as test room conditions, such as air mixing during testing. However, measurable reductions in bioaerosols, beyond natural airborne decay rate, were observed.

**Conclusion:**

Under the described test conditions, air cleaners containing high efficiency filtration significantly reduced bioaerosol levels. The best performing air cleaners could be investigated further with improved assay sensitivity, to enable measurement of lower residual levels of bioaerosols.

## Introduction

Air cleaning devices are intended to remove particulate material from the air, generally doing so in the background while the treated area is occupied. They differ in design and not all contain scientifically proven technology or provide relevant test data to support manufacturer claimed performance.^1^ The claims made for the benefits of air cleaners have become so widely publicized, especially in the context of the COVID-19 pandemic, that a rapid evidence review was commissioned by United Kingdom (UK) Government in late 2020, to consider the efficacy of various systems.^2^

The information from Scientific Advisory Group for Emergencies-Environment and Modelling Group (Sage-EMG) and Chartered Institution of Building Services Engineers^1,2^ emphasizes that air cleaners are most appropriate for spaces where there is insufficient ventilation and/or where existing ventilation cannot be improved. The Sage-EMG also concluded that air cleaning devices using germicidal UV and/or high efficiency air filtration are most likely to achieve safe and effective bioaerosol removal, but that other air cleaning systems, such as those emitting free radicals or ionizing effects, are not excluded from consideration. These other particulate removal methods are, however, deemed less likely to have the impact required to influence airborne disease transmission, based on data available at the time of that review.^2^

Air cleaners are typically left running for long periods in treated spaces and as such, they should not produce harmful chemical by-products.^3^ One way to assess their performance is by measuring their clean air delivery rate (CADR). This indicates how rapidly an air cleaner can remove airborne particles from the air of a room and the higher the CADR, the more particles the air cleaner will remove and the larger the room volume it can serve.^4–6^ Some available systems are scalable, and others can be inserted into existing ventilation ductwork.^1^ For the purposes of this study, only floor-standing or wall-mounted units were tested.

Most published air cleaner studies are small-scale assessments of one or two devices, with test conditions often differing greatly between studies, making comparisons of performance difficult. Some published reports are based on first principles, rather than scientific evidence.^7^ Others relate to non-biological air quality tests, rather than bioaerosols.^8,9^ In terms of expressing measured efficacy, some authors have used disease incidence or frequency of disease symptoms in a setting as a measurement method.^10,11^

Other reports present environmental contamination levels measured before and after air cleaner intervention.^9,12,13^ However, precise data about the ability of a device to reduce bioaerosol levels remain limited and there is little consistency across the design of investigative tests. At the time of writing, no internationally accepted test standard exists for evaluating air cleaning devices performance against bioaerosol challenge, even at the most basic level. Straightforward side-by-side comparisons of multiple systems are also rare.

Safety in use is an important consideration for these devices but despite their widespread marketing, relatively little safety information exists. A published guidance note to health care premises does describe conditions for safe operation.^14^ This includes the recommended use of respiratory protective equipment and/or personal protective equipment when filters are changed and the importance of following the manufacturer's instructions in relation to high efficiency particle arrestance (HEPA) filter/UV lamp replacement and any periodic cleaning of the air cleaner's interior surfaces.

Some reports have suggested ozone as a possible by-product, though this is dependent on the air cleaning mechanism used. Electrostatic particle precipitators are reported as those most likely to cause this by-product due to their use of corona discharge mechanisms.^15^ For other types of air cleaner the published information for ozone by-product is limited.^16^ Some air treatment devices do produce localized ozone by design, utilizing its biocidal effect, but these systems are generally based on the use of UV and ozone, have no HEPA filtration, and are outside the scope of this study.

The main aim of this study was to test and compare the performance of five air cleaning devices of standalone floor or wall-mounted design, incorporating either HEPA filtration only or HEPA filtration in combination with germicidal UV. The assessments were made using bacteriophage MS2 (hereafter known as MS2), as a bioaerosol challenge, with viable MS2 recovery assessed, alongside parallel total particle count data. MS2 is a single-stranded RNA virus that has been widely used as a mammalian virus surrogate and is known to survive well during aerosolization.^17–19^

The five tested devices were all monitored for ozone and hydrogen peroxide (H_2_O_2_) by-product generation using calibrated measurement sensors capable of parts per million (PPM) level detection or better.

## Methods

### Air Cleaner Systems Tested

For the purposes of this publication, we refer to the test equipment based on specification and design principles, rather than by brand name. The systems on test are described in Table 1 and all were run on their maximum flow rate during testing, if this feature was adjustable.

**Table 1.** Air cleaning equipment tested

Device ID reference	Mode of operation	Additional comments	Claimed air flow rate (if known)
#1	Combined high efficiency filtration and cold atmospheric pressure plasma unit	Small, portable unit. Floor or table top mounted. Brushed steel outer casing. Manual control. Dimensions: 0.19 × 0.35 × 0.28 m. Max. claimed noise level: 55 dB	Claimed max. 150 m^3^/h with the specification tested. Scalable, depending on application.
#2	Circular HEPA filter unit with radially dispersed clean air delivery.	Wall mounted. Combined powder-coated steel/plastic outer casing. Manual control. Dimensions: 0.2 × 0.5 × 0.5 m Max. claimed noise level: 72.5 dB	Claimed max. 600 m³/h
#3	HEPA EN 1822 H14 level air filtration unit with activated carbon filter.	Wall mounted with heavy duty powder-coated steel outer case. Remote control. Dimensions: 0.59 × 0.39 × 0.42 m. Max. claimed noise level: 58 dB.	Claimed max. 1440 m^3^/h with cyclonic air circulation, designed to mix room air. Scalable, depending on application.
#4	HEPA EN 1822 H13 with activated carbon filter and UV lamp emitting at 253.7 nm.	Floor-standing unit with lightweight metal case. Remote control. Dimensions: 0.43 × 0.13 × 0.72 m. Max. claimed noise level: 55 dB.	Claimed max. 380 m^3^/h
#5	PM20 grade cotton prefilter, HEPA filter with activated carbon filter. UV lamp emitting at 253.7 nm.	Small portable unit. Floor or tabletop standing. Remote control. Dimensions: 0.30 × 0.33 × 0.13 m. Noise level: not given.	Total air flow rate not provided but claimed CADR: 62 m³/h

CADR, clean air delivery rate; HEPA, high efficiency particle arrestance.

### Test Room Facility

All testing was carried out in a fully gas sealable exposure chamber of brushed stainless steel construction, with a 34 m^3^ room volume and temperature and humidity control stabilized at 22°C and 40% relative humidity (RH) respectively, before each test. A stationary air mixing fan, 0.8 m high, was used to ensure the uniform mixing of particles inside the chamber for the duration of each test. On completion of each test, the room was purged with clean air for 30 min, before staff re-entering the room to recover equipment and materials.

### Bioaerosol Generation

Bacteriophage MS2 (ATCC 15597-B1) was used as a bioaerosol test challenge for this work. Fresh cultures of MS2 were prepared from frozen bacteriophage stocks and propagated in *Escherichia coli* using nutrient broth (Oxoid) without supplements. Briefly, the preliminary *E. coli* culture (*E. coli* ATCC^®^ 15597) was prepared by adding 1 mL *E. coli* stock to 100 mL nutrient broth (1.3% w/v) and maintained at 37°C for 3–4 h in a shaking incubator at 70 rpm. A 100 μL volume of MS2 stock was added to the *E. coli* host culture and maintained overnight at 35°C in a shaking incubator at 70 rpm.

After incubation, the MS2 suspension was centrifuged at 3000 rpm (1690 *g*) for 10 min to remove host cell debris. The supernatant was filtered consecutively through 0.45 and 0.2 μm filters (Millipore, United Kingdom). To quantify the stock MS2 as plaque forming units per mL (PFU/mL), the freshly filtered stock was diluted using a 10-fold serial dilution in phosphate-buffered saline (PBS). One hundred microliters of each dilution was mixed with 300 μL *E. coli* (3–6 h culture). The MS2:*E. coli* mix was then added to 3 mL nutrient agar overlay and poured onto 1.5% Oxoid nutrient agar plates without supplements and incubated overnight at 35°C. PFU/mL of the MS2 stock was then back calculated. Forty milliliters volumes of MS2 were added to the Collison nebulizer before each test run.

A bioaerosol of MS2 was generated in the exposure chamber using a Collison 6-jet nebulizer,^20^ operated from breathing quality compressed air delivered at 25 psi for 30 min. This provides a nebulizer flow delivery rate of 12 L/min. The liquid bacteriophage suspension was prepared so as to sit at a starting level exactly 1 cm above the lower tip of the Collison inlet tube, as recommended by the supplier. An assessment of particle sizes generated by the Collison nebulizer, based on Optical Particle Sizer (OPS) measurement, confirmed that the majority of nebulized particles fell within 0.1–1.4 μm size range (data not shown).

This is consistent with the findings of others^21,22^ and is within the respirable particle size fraction, that is, if inhaled, a particle of this size may penetrate deep into the lungs, rather than depositing in the upper airways. It is known that the diameter of droplets generated by sneezing, coughing or speech may vary from <1 to 100 μm, but also recognized that those particles in the medium to large size range may either fall out of the airborne state, or else reduce in diameter due to rapid drying effects while in the airborne state.^23–25^ Based on these earlier reports, the particle sizes produced by the Collison nebulizer were deemed appropriate for this air cleaning challenge study.

### Bioaerosol Testing Procedure

Before bioaerosol generation, with the test chamber stabilized at a target temperature of 22°C and 40% RH, the stationary, oscillating air mixing fan was turned on to ensure uniform distribution of aerosols inside the chamber.

Twelve all glass impingers (AGI-30^26^), each containing the recommended volume of sterile PBS, were placed in the chamber at one collective location. These were used to sample the air sequentially at 12.5 L/min over four time points across a 3 h test period. The impingers were secured to their stands at worktop height. Vacuum pumps, positioned outside the test chamber, were connected to these via Teflon tubing running through a sealed port in the wall of the test room, allowing pump control from outside the test room. Three impingers were run simultaneously for 15 min at each time point as follows: immediately after aerosol generation (*T* = 0); after *T* = 1 h; *T* = 2 h and *T* = 3 h.

Using the earlier cited test conditions and sampling time points, three identical “baseline” control runs were undertaken, to assess the natural decay of the bioaerosol over 3 h with no air cleaner in the room. A fourth control run was later completed as a final check of sampling equipment performance. This was done before air cleaner testing and the baseline testing was therefore the only test sequence to include four test runs, rather than three. This was then followed by three repeat test runs for each of the five air cleaner devices.

A pre-calibrated optical particle scanner TSI_OPS (TSI, Buckinghamshire, UK) was used to log particle measurements over the test period, commencing at background levels just before activating the nebulizer, through to each run completion point. Particle count measurements were made for all control and test sampling runs.

### Sample Processing and Quantification

A fresh *E. coli* culture was prepared for each test run by adding 1 mL *E. coli* stock suspension (10^10^ to 10^11^ CFU/mL) to 100 mL nutrient broth and incubating at 37°C for 4 h at 70 rpm in a shaking incubator. Impinger PBS sample volumes were removed by pipetting, and the recovered volume was noted. Sample losses during impinger use were calculated, and fresh sterile PBS was added to replenish the original total volume for each impinger. The air sample suspensions and MS2 stock (as used in the Collison nebulizer) were each prepared as 10-fold serial dilutions. Each dilution of MS2 sample was added in 100 μL volumes to 300 μL of the 4 h *E. coli* culture.

The MS2/*E. coli* was then mixed with 3 mL of nutrient semi-solid overlay (maintained at 55°C until used) and immediately poured onto nutrient agar plates. Samples were left to cool and solidify for 20 min and incubated at 35°C for overnight. After incubation, plates were examined visually for PFU, were counted and log reduction was calculated.

### Ozone and H_2_O_2_ by-Product Measurement Close to Air Cleaners

Ozone levels were measured using a 2B Technologies Model 205 Dual Beam Ozone Monitor, accurate to within 1 part per billion (ppb). The pre-calibrated monitor was located outside of the test room, and appropriate diameter Teflon™ tubing was run in to the test area via an access port, with the tip of the tubing placed within 10 cm of the outlet of the air cleaner. Ozone levels were logged constantly, for the duration of each test. H_2_O_2_ levels were measured with an ATI Portasens III D16 portable gas detector fitted with a newly calibrated low level H_2_O_2_ sensor, capable of detecting to 0.1 ppm. The whole monitor was placed with its sampler tip within 10 cm of each device during air cleaner operation. The UK workplace exposure limit for both chemicals falls within the low ppm range.^22^

### Statistical Analysis

The microorganism concentration used for analysis was the mean of the air samples taken at each time point (*T* = 0 h, *T* = 1 h, *T* = 2 h, *T* = 3 h) across the multiple test runs. The bacteriophage impinger-based sampling method had a lower limit of detection (LOD) of 200 PFU/m^3^ of sampled room air. Therefore, an observed value of zero PFU of air might not represent a true zero. To account for this, a ½ LOD value was used in place of a zero reading at sample level, before taking the mean of the three samples at each sampling time point.

Reduction in microorganism levels after 3 h of air cleaning treatment was measured using log reduction analysis over the test period, which was calculated as log_10_ (untreated air concentration) minus log_10_ (treated air concentration). As there were three runs for each air cleaning system, there are three log reduction figures and log_10_ (untreated air concentration) figures for each system. These data are best summarized by showing the range of the values obtained.

The variation in log reduction and log concentration with time was also determined for each system and plotted on graphs to allow a comparison of the systems. These figures were obtained by calculating the mean concentration at each time point across all samples and all runs for each system and then taking the log value. Thus, each point plotted is a log of the mean, rather than a mean of the logs.

The CADR combines the effects of filtration efficiency and the effectiveness of the air cleaner to draw the test chamber's air through it.^4,5^ CADR, therefore, provides an indication of comparable performance between different air cleaning systems. Foarde^4,5^ refers to CADR as CARm when it is applied to microbiological (bio)aerosols, and provides a formula for calculating CARm, which has been applied here in a slightly amended form to derive a metric (rather than imperial) measure:

$$CARm=60V\left(k_{e}-k_{n}\right)$$

where: *V* is the volume of the test chamber in m^3^; *k_e_* is the measured particulate decay rate (min^−1^) for the bioaerosol; *k_n_* is the natural decay rate (min^−1^) for the bioaerosol; The expression is multiplied by 60 so that CARm is measured in units of m^3^/h.

The particulate decay rate is defined as the decrease in particles while the air cleaner is operating, whereas the natural decay rate is the decrease when an air cleaner is not used. The work of Foarde^4,5^ provides the formula that is used to calculate decay rate.

The CARm calculations were performed twice; first using the culture-based PFU data, and second using OPS particle counts, which were total counts of all particles within the test room air. In calculating CARm, the concentration of bioaerosols at all available time points was used. As the PFU concentration was measured at *T* = 0 h and subsequently every hour for 3 h, the PFU dataset comprises four time points in each run that were used to calculate CARm. This was in contrast to the calculation of log reduction, where only the initial and final time points were used.

The OPS particle counts covering a wide range of particle sizes were logged, reflecting the range of particles present in the test room after bioaerosol generation. The Collison nebulizer produces particles mainly within the 1–10 μm range, and where particles of <1 μm are present, these are counted less reliably by the OPS. For this reason, particles of <1 μm were excluded from the OPS data analysis. The OPS allocates counted particles in to “bins” based on their size, and the closest bin to 1 μm had a minimum threshold of 1.117 μm, so all particles of this size or larger were included. These particles were summed at each time point.

For the purposes of calculating CARm, OPS data were used from the point at which aerosol generation was at its peak (immediately after the aerosol generator was stopped), until completion of the *T* = 3 h triplicate air samples. Any values before or after these periods were excluded as they did not relate to the monitored bioaerosol decay period.

For both CARm calculations (using PFU and OPS counts), the decay rate was calculated for each run, and the mean rate obtained for each system, before calculating CARm and its standard error. The formula for the standard error of the CARm was derived here from the standard errors of the decay rates and the formula for CARm above. For example, for air cleaner #4:

$$\begin{array}{cc} & {Standarderrorofmeanbaselinedecayrate,SE}_{B}\\ & =\sqrt{\frac{Varianceofbaselinedecayrate}{Numberofbaselineruns\left(4\right)}}. \end{array}$$

$$\begin{array}{cc} & {Standarderrorofmeanaircleaner\#4decayrate,SE}_{N}\\ & =\sqrt{\frac{Varianceofaircleaner\#4decayrate}{Numberofaircleaner\#4runs\left(3\right)}}. \end{array}$$

$$\begin{array}{cc} & StandarderrorofCARmestimatefor\#4\\ & =60V\sqrt{\left({SE}_{B}^{2}+{SE}_{N}^{2}\right)}, \end{array}$$

where *V* is the air volume of the test chamber in m^3^.

## Results

### Log Reduction

The range of log reduction results obtained after a 3 h test period and log_10_ (untreated air concentration) for each system across the experimental runs are shown in Table 2.

**Table 2.** Range of log reduction values and the range of log(untreated air concentration) values

	Log reduction range		Log(untreated air concentration) range^**a**^	
System identifier	Minimum	Maximum	Minimum	Maximum
Baseline^b^	0.12	1.55	7.59	9.90
#1	3.14	3.37	5.99	7.30
#2	4.03	5.41	8.14	8.64
#3	3.13	5.39	7.24	8.54
#4	4.71	5.31	7.44	8.86
#5	1.91	2.37	7.89	8.61

Log(untreated air concentration) varies for each system and each run and represents a “ceiling” for maximum log reduction that could be achieved.

^a^ This range represents the bioaerosol concentrations immediately after the generation of the bioaerosol atmosphere but before air cleaner was switched on for systems #1 to #5.

^b^ Natural bioaerosol decay range observed over 3 h and based on four control runs, with no air cleaner in room.

The untreated concentration of bioaerosol in the room at the start of each test represents the maximum theoretically achievable log reduction, that is, bioaerosol levels present in the room air just as bioaerosol generation was stopped and air cleaning commenced. Bioaerosol reduction exceeding natural decay was observed for all air cleaners. Reductions varied between devices from <2-log for the least effective, to a >5-log reduction for the three most efficacious systems (Table 2).

In Table 2, performance differences can be seen for systems 1 and 4. For a well-performing air cleaner, a greater potential for bioaerosol reduction is best demonstrated when the bioaerosol challenge concentrations are higher. Table 2 shows that the starting concentrations for system 1 were lower at the top and bottom of the indicated range compared with systems 2, 3, and 4, despite efforts to maintain bioaerosol challenge consistency across tests.

This may have limited the demonstrable performance of system 1 compared with other air cleaners. Conversely, systems 2 and 5 had very similar bioaerosol starting concentrations, but showed quite different overall log reductions, and here it is clear that system 5 performed less effectively than system 2.

Full bioaerosol removal was never achieved for any of the test systems (Table 2). For some systems, this was where a zero PFU observation was observed after treatment, but where the assay LOD adjustment meant that a PFU figure above zero was substituted instead. This may have underestimated the actual removal efficiency of some air cleaning systems.

Also in Table 2, the untreated bioaerosol baseline value represents a worst-case scenario in terms of bioaerosol decline as this is where no air cleaner is used and only the natural airborne decay of the MS2 challenge was recorded. Table 2 shows that test system 5 was the least effective of the systems tested, as it gave low levels of log reduction that were substantially below the other systems on test. From Table 2, systems 2, 3, and 4 all gave higher log reductions than system 1, but they also had higher untreated (starting) concentrations.

System 4 performance appeared better than the other systems. However, three data points were recorded for each test system, and at each time point a substantial spread of bioaerosol reduction values was observed despite the consistently applied test approach. This was especially true for system 3, and more data would be needed to unequivocally confirm which system (2, 3 or 4) offered the best levels of log reduction overall, based on PFU counts.

**Figure 1.**
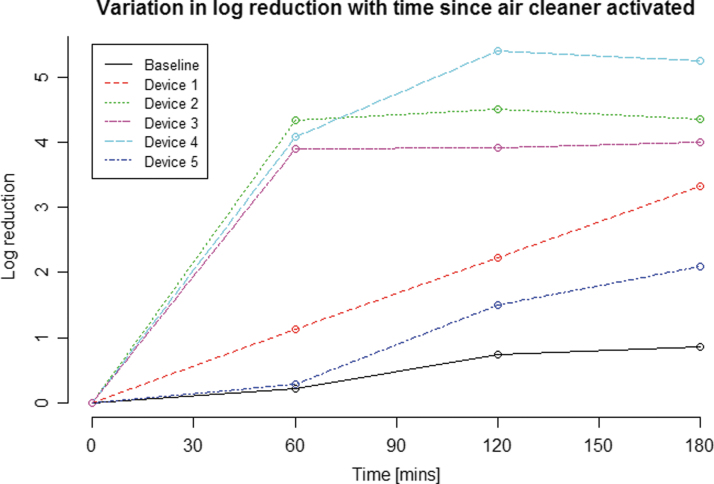
Observed log reduction after 3 h and log (untreated air concentration) values by system and run. The log reduction at each time point after the air cleaner is activated is shown. The log reduction here is derived from the log of the mean of the MS2 bacteriophage concentrations across the three runs for each system (four runs for untreated baseline air).

The change in log reduction over time, after the activation of each air cleaner, is shown in Figure 1. The data were derived from the log of the mean of the three test run concentrations obtained at each time point. Each system is represented by a different color and a different line type. As would be expected, the baseline data (no air cleaner) display the lowest rate of log reduction. The worst performing air cleaner system was system 5.

Of systems 1 to 4, three gave substantially higher log reductions (of around 4 logs) during the first hour, whereas the fourth system (system 1) was less effective, with a log reduction of only 1.1 logs after the first hour. However, system 1's log reduction performance continued to increase in a linear manner throughout the test period, reaching 3.3 logs after 3 h. The performance of systems 2 and 3 leveled off after the first hour, so that their log reductions were 4.4 and 4.0 logs respectively after 3 h. System 4 performance leveled off only after 2 h, achieving a bioaerosol reduction of 5.3 logs after 3 h. Based on Figure 1, system 4 was the best performing air cleaner of those tested.

**Figure 2.**
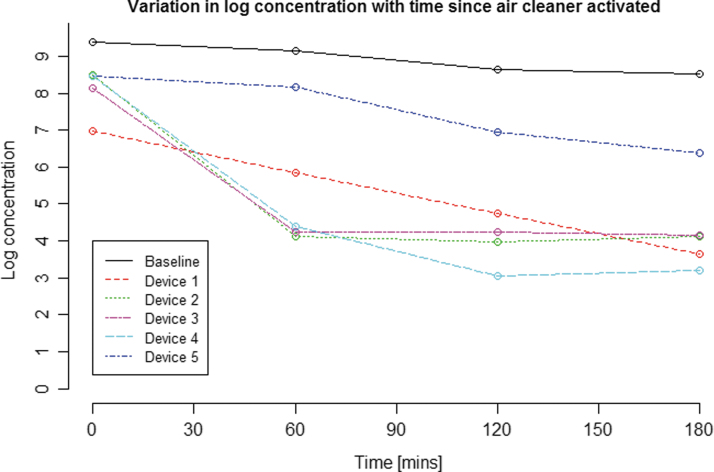
Log concentration at each time point after the air cleaner is activated. The log concentration here is the log of the mean of the MS2 bacteriophage concentration across the three runs for each system (four runs for untreated baseline air).

Table 3 shows the log reduction achieved for each system after 3 h alongside values for the untreated concentration. These data also show the log reduction as a percentage of the “ceiling,” another indication of overall system performance, with system 4 again giving the greatest impact by this measure, system 5 the least. Figure 1 presents these data visually, showing the progressive log reduction for each system over time, with Figure 2 providing a further comparison of system performances, showing variation in log concentration over time.

**Table 3.** Log reduction value after 3 h and showing the “ceiling” log reduction

	Log reduction after 3 h			Log reduction (as percentage of “ceiling”)	
System	Zeroes replaced with ½ LOD value	Include zero counts	“Ceiling” log reduction	Zeroes replaced with ½ LOD value (%)	Include zero counts (%)
Baseline	0.85	0.85	9.38	9	9
1	3.32	3.36	6.99	48	48
2	4.36	4.36	8.48	51	51
3	4.00	4.00	8.15	49	49
4	5.25	5.34	8.46	62	63
5	2.09	2.09	8.45	25	25

The log reduction is derived from the log of the mean of the concentrations across the three runs for each system (four runs for baseline). Two scenarios are included: the first where recorded zero PFU counts are substituted with ½ LOD values; the second where the zero counts are included and not replaced.

LOD, limit of detection; PFU, plaque forming units.

Despite a number of zero PFU counts being recorded over the course of all testing (17 zero counts from a total of 45 mean PFU counts obtained during air cleaners use; data not shown), Table 3 confirms that the adjustment of zero counts using ½ LOD values had only a small effect on calculated log reduction. Data obtained with or without a zero count adjustment demonstrated the same trends and very similar measurement values for each tested air cleaner.

### Clean Air Delivery Rate

The calculations of CARm based on PFU concentrations are shown in Table 4 in descending order of efficacy and show that system 5 was the least effective system tested. This was consistent with calculations based on bioaerosol log reduction data, with system 1 the next worst performer. Data in Table 4 show that system 4 had the highest CARm based on PFU concentrations. However, the relatively high standard errors for the three units with the highest CARm values mean it is not possible to conclude from these data which was the best performing system. System 3 had a particularly high standard error, and this was consistent with the observations in Table 2, as influenced by its performance during test run 1, which was better than its second and third test runs.

**Table 4.** Clean air delivery rate based on plaque forming units concentrations—shown in descending order of performance

System	CADR (m^3^/h)	Standard error
4	98.0	10.2
2	86.0	11.1
3	77.3	17.7
1	57.4	6.7
5	34.6	6.9

A decay rate was obtained for each run. The CARm for each system (and its standard error) is derived from these figures.

An Analysis of Variance test found that there was a highly significant difference in the system decay rates underlying these CARm values (*p*-value = 0.006). A Tukey multiple comparison of means (with a 95% family-wise confidence level) showed that none of the system decay rates were found to be significantly different from each other (adjusted *p*-value >0.05), with the following exceptions: systems 5 and 2 were significantly different (adjusted *p*-value = 0.021) as were systems 5 and 4 (adjusted *p*-value = 0.006). Importantly, when comparing the decay rates of the best two performers (4 and 2), the adjusted *p*-value was 0.89.

The calculation of CARm based on OPS counts (Table 5) found that system 1 gave a low CARm value. This was surprising, given the system's better performance based on PFU concentrations, and implies that the OPS data for system 1 may have been skewed. It was noted that the raw OPS particle data for system 1 contained a high number of coincidence error readings (data not shown). This occurs when bioaerosol levels exceed the upper accurate detection range for the OPS and would have influenced OPS particle detection accuracy. Of the remaining systems, system 5 had the next lowest CARm, with a similar value to that obtained using PFU concentrations.

**Table 5.** Clean air delivery rate based on total optical particle sizer particle counts (across all bins of size ≥1.117 μm)—shown in descending order of performance

System	CADR (m^3^/h)	Standard error
3	435	16.6
2	334	3.8
4	281	11.2
5	29.1	4.1
1	4.0	4.0

A decay rate was obtained for each run. The CARm for each system (and its standard error) is derived from these figures.

The CARm values calculated for the three remaining systems (system 2, 3, and 4) were all more than double those based on PFU concentrations (Table 4) and the order of performance was reversed, so that system 3 gave the highest CARm and system 4 the lowest CARm of these three systems.

### Ozone and H_2_O_2_ Measurement

Only one system (#1) generated detectable airborne ozone emission during sealed room chamber testing, with a maximum 550 ppbv (0.55 ppmv) detected. This exceeds the UK workplace exposure limit for ozone gas.^27^ However, it was recognized that a sealed 34 m^3^ test environment was not representative of how the unit would be used in the real world. Its residual ozone production was, therefore, tested again in a similarly sized laboratory space with a mechanical ventilation rate confirmed at 8 air changes per hour. Under these conditions, no ozone was detectable. Similarly, there was no detectable evidence of H_2_O_2_ residue in the air within the test environment for any of the five air cleaners, even with the room sealed for experimental purposes.

## Discussion

All systems tested here met the criteria for an air cleaner system that included high efficiency filtration and could treat the air of a room of dimensions used (34 m^3^). All five test systems demonstrated the ability to remove MS2 bioaerosols successfully from the test room environment, exceeding losses by natural decay alone, though considerable performance variation was observed across the equipment tested. It was always likely that such differences would be observed, since the test systems covered a range of claimed flow rates and system designs.

System 5 consistently gave the weakest performance, in terms of both log reduction (compared with maximum potential) and CADR. This coincided with the lowest claimed flow rate of the five systems so it was not surprising. System 4 gave the best log reduction profile with time. It was one of three systems giving a log reduction of around 4 logs after the first hour of operation. However, unlike systems 2 and 3, there was a further substantial improvement in log reduction during the second hour of operation for system 4, which was still providing the best log reduction value of all the systems tested during the later stages of the tests.

Even when the maximum achievable log reduction (based on starting concentration) was taken into account by calculating log reduction percentage, system 4 was the best performer. System 4 is a combined HEPA/UV-C treatment system and despite its overall performance, it did not have the highest claimed flow rate of the five systems, based on supplier information.

Four of the five test systems yielded some zero detectable PFU counts during testing. The LOD of the bacteriophage assay meant that this did not necessarily indicate that all MS2 bacteriophage had been removed from the test room air. The effect of adjusting the zero value using a ½ LOD value had little effect on log reduction results, with each system's performance trends unchanged.

Although three systems provided higher log reduction performances than system 1 over the 3 h test period, system 1 performance did not level off in the manner of systems 2, 3, and 4. Bioaerosol log reduction using system 1 increased at a constant rate, and thus we might speculate that if the test period had been longer, system 1's performance might have exceeded that of the other systems. Only a longer test period could confirm this, and this was beyond the scope of the current study.

When CADR was calculated using PFU concentrations, there was a highly significant difference in the system decay rates underlying these CARm values. System 4 gave the best performance, but there was not a statistically significant difference between this and the next best performer, system 2 or any of the other systems, apart from system 5.

The calculation of CARm based on OPS counts found that system 1 gave an unexpectedly poor CARm value. This is surprising given the system's performance based on PFU concentrations, and it implied that OPS data for system 1 were in some way inaccurate. This may have occurred because the OPS data for system 1 were found to have numerous “coincidence errors,” which reflect the OPS trying to measure aerosol levels beyond its calibrated range.

If particle concentration is increased sufficiently, more than one particle occupies the same space in the OPS sensing volume and “coincidence” occurs, resulting in a drop in counting efficiency and particle sizing inaccuracy.^28^ This can cause inconsistencies in OPS performance and may explain why the OPS CARm value was not as high as expected for system 1. Of the remaining systems, system 5 gave the next lowest CARm, with a similar value to that obtained using PFU concentrations.

However, the CARm values calculated for the three remaining systems (2, 3, and 4) were all more than double those based on their PFU CARm performance. Based on OPS particle counts, the order of performance was therefore reversed, so that system 3 gave the highest CARm and system 4 the lowest value of these three systems.

The OPS counts and associated CARm values clearly indicate that system 3 was the best performing machine, despite the high standard error of its estimated CADR. The authors are aware of a specific recommendation for system 3 operation that may have influenced its performance. In operating this device, the manufacturer recommends it be allowed to run and acclimatize for ∼30 min before testing as opposed to turning the device on immediately after bioaerosol chamber release.

To keep test conditions consistent with other systems and to allow bioaerosol delivery into the chamber immediately before air cleaner activation, this device acclimatization was not possible. This may explain some inconsistencies in the performance of system 3.

The PFU log reduction figures conclusively show system 4 as the best performing system. However, the relative performance of the different systems based on CARm varies substantially according to the type of count data used, so it is not possible to conclude which is the best system tested overall, based on PFU and OPS counts. What is clear is that systems 2, 3, and 4 are consistently the strongest performers and these devices are based on HEPA, HEPA, and HEPA/UV-C respectively.

Airflow is clearly important to the performance of air cleaners, but the results from air cleaner 4, which did not have the highest claimed airflow value (Table 1), demonstrate that airflow rate alone is not the whole story. Other factors may come in to play, such as the internal design of the air cleaner systems, including the size and quality of the installed HEPA filter(s) and related filter fit/seating within the machine.

Also, the presence/absence of additional elements such as coarse filters/germicidal UV lamps/physical baffling to slow air flow down over UV emitters. During this study, we used the air cleaners as received and did not open them up to see how well they were put together internally, so such influences were outside the scope of our investigation.

## Conclusion

To date, there is little information and a lack of consistent research regarding the ability of air cleaning technologies to remove bioaerosols in a side-by-side comparison study. This work reports a comparison study using a selection of air cleaning devices, which were either portable/floor standing or wall mounted, to determine their efficiency against a biological challenge in combination with CARm assessment. Under the described conditions, all the selected air cleaners provided some reduction in the bioaerosol concentration.

These devices were evaluated using high bioaerosol challenge concentrations, probably far higher than most real-world situations would create. Some devices performed well under these challenging conditions, reducing the concentration below the experimental detection threshold. To determine more definitive CARm, the best performing air cleaners could be investigated further with improved assay sensitivity, to enable measurement of lower residual levels of bioaerosols.

Overall, when considering the high level of biological challenge, the worst performing device still demonstrated a measurable reduction in bioaerosol concentration. This suggests that in real-world scenarios, even the worst performing device could potentially help to mitigate bioaerosol spread and may offer benefits for reducing the airborne spread of viruses such as SARS-CoV-2.

## References

[1] Chartered Institute of Building Services Engineers (CIBSE). COVID-19: Air Cleaning Technologies (v1). 2021. Available from: https://www.cibse.org/knowledge-research/knowledge-portal/covid-19-air-cleaning-technologies-v1 [Last accessed: June 27, 2022].

[2] The Scientific Advisory Group for Emergencies (Sage)—Environment and Modelling Group. Rapid Review of Air Cleaning Devices; 2020. Available from: https://www.gov.uk/government/publications/emg-potential-application-of-air-cleaning-devices-and-personal-decontamination-to-manage-transmission-of-covid-19-4-november-2020 [Last accessed: June 27, 2022].

[3] Collins DB, Farmer DK. Unintended consequences of air cleaning chemistry. Environ Sci Technol 2021;55(18):12172–12179 34464124 10.1021/acs.est.1c02582

[4] Foarde KK. Methodology to perform clean air delivery rate type determinations with microbiological aerosols. Aer Sci Technol 1999;30(2):235–245. https://www.tandfonline.com/doi/abs/10.1080/713834074

[5] Foarde KK. Development of a method for measuring single-pass bioaerosol removal efficiencies of a room air cleaner. Aer Sci Technol 1999;30(2):223–234; doi: 10.1080/027868299304804

[6] US-Environmental Protection Agency. Guide to Air Cleaners in the Home, 2nd edition; 2018. Available from: https://www.epa.gov/sites/default/files/2018-07/documents/guide_to_air_cleaners_in_the_home_2nd_edition.pdf [Last accessed: June 27, 2022].

[7] Elias B, Bar-Yam Y. Could Air Filtration Reduce COVID-19 Severity and Spread. New England Complex Systems Institute. 2020;9. Available from: https://necsi.edu/could-air-filtration-reduce-covid19-severity-and-spread. [Last accessed: June 27, 2022].

[8] Offermann FJ, Sextro RG, Fisk WJ, et al. Control of respirable particles in indoor air with portable air cleaners. Atmosph Environ 1985;19(11):1761–1771; doi: 10.1016/0004-6981(85)90003-4

[9] Du L, Batterman S, Parker E, et al. Particle concentrations and effectiveness of free-standing air filters in bedrooms of children with asthma in Detroit, Michigan. Build Environ 2011;46(11):2303–2313; doi: 10.1016/j.buildenv.2011.05.012 21874085 PMC3161201

[10] Verhougstraete M, Reynolds K. Use of a portable air disinfecting system to remove seeded coliphage in hospital rooms. Am J Inf Control 2016;44(6):714–715; doi: 10.1016/j.ajic.2015.12.025 26905789

[11] Rao NG, Kumar A, Colon C, Goswami DY. Impact of a new portable air purification technology device in the pediatric hospital setting—A pre-post assessment study. Cureus 2020;12(3); doi: 10.7759/cureus.7440 PMC7186091 32351820

[12] Boswell TC, Fox PC. Reduction in MRSA environmental contamination with a portable HEPA-filtration unit. J Hosp Inf 2006;63(1):47–54; doi: 10.1016/j.jhin.2005.11.011 16517004

[13] Brągoszewska E, Bogacka M, Pikoń K. Efficiency and eco-costs of air purifiers in terms of improving microbiological indoor air quality in dwellings—A case study. Atmosphere 2019;10(12):742; doi: 10.3390/atmos10120742

[14] Scott J, Zanoni PG. Michigan Department of Licensing & Regulatory Affairs (LARA)—Guidelines for use of Portable Air Filtration Systems in Health Care Facilities; 2002. Available from: https://www.michigan.gov/documents/mdch/bhs_hepa_337337_7.pdf [Last accessed: June 27, 2022].

[15] Lee Y, Kim YS, Han B, et al. Minimizing the size and ozone emission of electrostatic precipitators using dielectric and rolled carbon film coatings. IEEE Trans Ind Appl 2021;58(1):753–759; doi: 10.1109/TIA.2021.3122402

[16] Sublett JL. Effectiveness of air filters and air cleaners in allergic respiratory diseases: A review of the recent literature. Curr Allergy Asthma Rep 2011;11(5):395–402; doi: 10.1007/s11882-011-0208-5 21773748 PMC3165134

[17] Walls HJ, Ensor DS, Harvey LA, et al. Generation and sampling of nanoscale infectious viral aerosols. Aerosol Sci Technol 2016;50(8):802–811; doi: 10.1080/02786826.2016.1191617

[18] Dietrich WL, Bennett JS, Jones BW, et al. Laboratory modeling of SARS-CoV-2 exposure reduction through physically distanced seating in aircraft cabins using bacteriophage aerosol—November 2020. Morb Mortal Wkly Rep 2021;70(16):595; doi: 10.15585/mmwr.mm7016e1 PMC8061797 33886531

[19] Luo H, Zhong L. Ultraviolet germicidal irradiation (UVGI) for in-duct airborne bioaerosol disinfection: Review and analysis of design factors. Build Environ 2021;197:107852; doi: 10.1016/j.buildenv.2021.107852 33846664 PMC8021448

[20] CH Technologies. Collison Nebulizer— Instructions: MRE 1, 3, 6 and 24 Jet; 2016. Available from: https://chtechusa.com/Manuals/MRE_Collison_Manual.pdf [Last accessed: June 27, 2022].

[21] Paton S, Clark S, Spencer A, et al. Characterisation of particle size and viability of SARS-CoV-2 aerosols from a range of nebuliser types using a novel sampling technique. Viruses 2022;14(3):639; doi: 10.3390/v14030639 35337046 PMC8950415

[22] Crosswy FL. Particle Size Distributions of Several Commonly Used Seeding Aerosols. NASA. Langley Research Center Wind Tunnel Seeding Systems for Laser Velocimeters; 1985. Available from: https://ntrs.nasa.gov/citations/19860001974 [Last accessed: June 27, 2022].

[23] Han ZY, Weng WG, Huang QY. Characterizations of particle size distribution of the droplets exhaled by sneeze. J Royal Soc Interface 2013;10(88):20130560; doi: 10.1098/rsif.2013.0560 PMC3785820 24026469

[24] Gorbunov B. Aerosol particles generated by coughing and sneezing of a SARS-CoV-2 (COVID-19) host travel over 30 m distance. Aerosol Air Qual Res 2021;21(3):200468; doi: 10.4209/aaqr.200468

[25] Stadnytskyi V, Anfinrud P, Bax A. Breathing, speaking, coughing or sneezing: What drives transmission of SARS-CoV-2? J Int Med 2021;290(5):1010–1027; doi: 10.1111/joim.13326 PMC8242678 34105202

[26] May KR, Harper GJ. The efficiency of various liquid impinger samplers in bacterial aerosols. Br J Ind Med 1957;14(4):287; doi: 10.1136/oem.14.4.287 13471876 PMC1037828

[27] Health and Safety Executive (GB). EH40/2005 Workplace Exposure Limits Containing the List of Workplace Exposure Limits for Use with the Control of Substances Hazardous to Health Regulations 2002 (as amended); 2020. Available from: https://www.hse.gov.uk/pubns/books/eh40.htm [Last accessed: June 27, 2022].

[28] TSI. Optical Particle Sizer Spectrometer 3330—Operation and Service Manual. P/N 6004403, Revision F; 2013. Available from: https://www.kenelec.com.au/wp-content/uploads/2016/06/TSI_3330_Opticle_Particle_Sizer_Manual.pdf [Last accessed: June 27, 2022].

